# Sequential Helical–Axial–Helical Triple-Rule-Out CT Angiography: Technical Feasibility and Territory-Specific Image Quality in the Emergency Department

**DOI:** 10.3390/jcm15124640

**Published:** 2026-06-15

**Authors:** Yeon-Jun Kim, Gi-Yong An, Sung-Jin Cha, Sung Min Ko

**Affiliations:** 1 Department of Human Health Convergence, Graduate School, Kangwon National University, Samcheok 25913, Republic of Korea; yunsu202@naver.com; 2 Department of Radiology, Wonju Severance Christian Hospital, Wonju College of Medicine, Yonsei University, Wonju 26426, Republic of Korea; koreacurry@naver.com (G.-Y.A.); cktjdwls@naver.com (S.-J.C.)

**Keywords:** triple-rule-out CT angiography, emergency department, coronary CT angiography, pulmonary embolism, acute aortic syndrome, sequential acquisition

## Abstract

**Background/Objectives:** Triple-rule-out CT angiography (TRO-CTA) enables simultaneous evaluation of coronary, pulmonary, and aortic causes of acute chest pain, but conventional single-acquisition protocols may compromise vascular enhancement because of conflicting contrast timing requirements. This study evaluated whether a physiology-based sequential helical–axial–helical acquisition strategy could provide consistent tri-territory enhancement in emergency settings. **Methods:** In this retrospective single-center study, 71 consecutive evaluable emergency department patients (mean age, 66.6 ± 17.0 years; 33 women) with undifferentiated acute chest pain underwent TRO-CTA using a structured sequential protocol (pulmonary, coronary, and aortic phases) guided by individualized test-bolus timing. Objective image quality was assessed using vascular attenuation, signal-to-noise ratio (SNR), and contrast-to-noise ratio (CNR); subjective image quality was independently graded by two radiologists. **Results:** Mean vascular attenuation exceeded predefined diagnostic thresholds in all territories (pulmonary 546.7 ± 237.8 HU [95% CI, 490.4–603.0]; coronary 438.8 ± 113.9 HU [95% CI, 411.9–465.8]; aortic 604.3 ± 190.9 HU [95% CI, 559.2–649.5]). Diagnostic interpretability was achieved in all three territories in every technically analyzable examination without repeat contrast-enhanced imaging. Median subjective image-quality scores were 5 (IQR, 4–5) for pulmonary, 4.5 (IQR, 4–5) for coronary, and 4 (IQR, 4–5) for aortic phases; interobserver agreement was good to excellent. Mean total DLP was 461.5 ± 122.5 mGy·cm. **Conclusions:** A sequential physiology-based TRO-CTA strategy is technically feasible in a tertiary emergency setting and provides consistent tri-territory enhancement. Because this was a single-arm technical validation study, prospective comparative and outcome-based studies are required to confirm its clinical impact.

## 1. Introduction

Acute chest pain is one of the most common and diagnostically challenging presentations in the emergency department (ED). Although many patients ultimately have non-life-threatening conditions, timely exclusion of three potentially fatal entities—acute coronary syndrome (ACS), acute pulmonary embolism (APE), and acute aortic syndrome (AAS)—is essential for appropriate triage and management. Current chest pain pathways emphasize structured clinical risk assessment using electrocardiography (ECG), serial cardiac biomarkers, and selective imaging, with coronary CT angiography (CCTA) playing an important role in appropriately selected patients when ACS cannot be reliably excluded after initial evaluation [1,2,3,4].

According to current clinical guidelines, CT is central to the evaluation of the three major life-threatening causes of acute chest pain in appropriately selected patients. CCTA is recommended in low-to-intermediate-risk patients when ACS cannot be reliably excluded after initial evaluation [2,3,4], CT pulmonary angiography (CTPA) is widely used for suspected APE in clinically appropriate patients [5], and contrast-enhanced thoracic CTA is a first-line imaging test for suspected AAS because of its speed, availability, and ability to define the extent and complications of disease [6,7].

AAS is a spectrum of disorders caused by disruption of aortic wall integrity and includes classic aortic dissection, intramural hematoma, and penetrating atherosclerotic ulcer. Although these entities share overlapping clinical features, management and prognosis differ substantially; therefore, rapid imaging-based differentiation is essential [6,7].

TRO-CTA was developed as a comprehensive imaging approach to evaluate the coronary arteries, pulmonary arteries, and thoracic aorta within a single examination [8,9,10,11,12,13,14]. Despite its conceptual appeal, early-generation TRO-CTA protocols were limited by heterogeneous vascular enhancement, increased radiation exposure associated with retrospective ECG gating, and relatively high contrast media requirements [11,12,13,14]. Conventional TRO-CTA protocols are typically based on single-acquisition strategies, which require compromise in contrast timing among pulmonary, coronary, and systemic circulations. This limitation may result in suboptimal or heterogeneous enhancement in at least one vascular territory, particularly in emergency settings with variable hemodynamics.

Recent advances in CT technology—including wide-area detector systems, flexible acquisition modes, individualized contrast timing, and advanced reconstruction—have expanded the technical possibilities of cardiothoracic imaging [15,16,17,18,19,20,21]. These developments have renewed interest in structured sequential acquisition strategies that align scan timing with the distinct hemodynamics of pulmonary, coronary, and aortic circulations. Such approaches may enable phase-specific optimization of vascular enhancement without requiring additional contrast administration. However, evidence regarding the feasibility and consistency of sequential TRO-CTA protocols in real-world emergency populations remains limited [22,23].

Our institution is a tertiary academic center serving as a regional cardiovascular emergency referral facility. As a result, ED patients frequently present with advanced age, multiple comorbidities, and a relatively high pretest probability of significant cardiothoracic disease. Therefore, the purpose of this study was to evaluate the technical feasibility and territory-specific image quality of a structured sequential helical–axial–helical TRO-CTA protocol implemented under real-world tertiary ED conditions. This investigation was designed as a technical validation study rather than a diagnostic accuracy study.

## 2. Materials and Methods

This retrospective, single-center study was approved by the Institutional Review Board (IRB approval number: CR324049; approved on 2 July 2024), and the requirement for informed consent was waived.

### 2.1. Study Population

We retrospectively identified 90 consecutive adult patients who underwent TRO-CTA in the ED between July 2023 and March 2024 for evaluation of acute chest pain. TRO-CTA was performed in patients with undifferentiated symptoms in whom ACS, APE, and AAS were all considered clinically plausible after initial evaluation, including ECG, cardiac biomarkers, and clinical risk stratification, when these conditions could not be reliably excluded.

For territory-specific quantitative analysis, examinations were required to meet predefined evaluability criteria in all three vascular phases. Nineteen patients were excluded from the primary tri-territory quantitative analysis because at least one phase was technically limited, most commonly owing to patient motion, clinically significant arrhythmia, or inability to cooperate in the emergency setting. These exclusions were not based on final diagnosis, disease severity, or clinical outcome. Importantly, the excluded examinations should not be interpreted as protocol failures; they underwent the complete TRO-CTA protocol and were excluded only from formal per-territory quantitative analysis because one or more vascular territories failed predefined quantitative evaluability criteria. In most cases, at least one or two vascular territories remained clinically interpretable. Thus, the 21% exclusion rate reflects the real-world technical challenges of multiphase ECG-gated cardiothoracic CT in a tertiary ED rather than preselection of clinically favorable cases.

**Figure 1:**
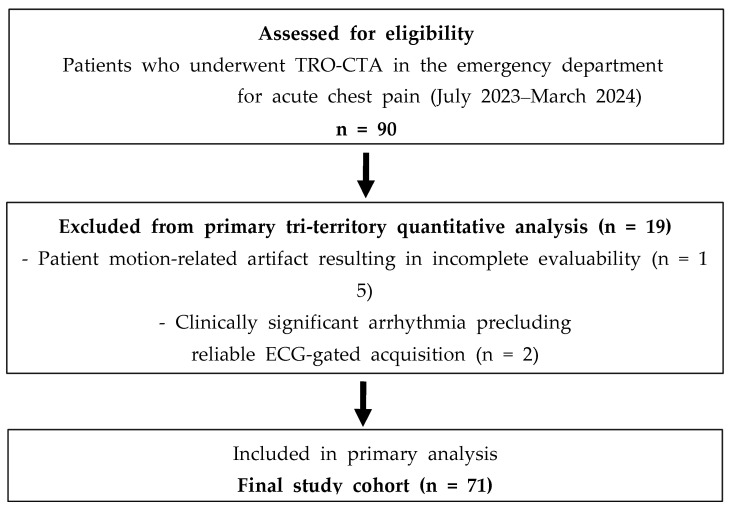
Flowchart of patient selection. Ninety patients who underwent emergency department triple-rule-out CT angiography (TRO-CTA) were retrospectively identified. Nineteen patients were excluded from the primary tri-territory analysis due to incomplete evaluability, most commonly related to patient motion (*n* = 15). The final study cohort consisted of 71 patients.

Patients meeting predefined exclusion criteria before imaging (e.g., contraindication to iodinated contrast material, prior contrast-enhanced chest CT for the same episode at an outside institution, or a definitive diagnosis established before TRO-CTA) did not undergo TRO-CTA and were therefore not included in the study cohort. The final study cohort for quantitative tri-territory analysis consisted of 71 technically analyzable examinations (Figure 1). No separate excluded-patient outcome or diagnostic table was added, because such analysis would exceed the technical feasibility scope of the present study and could imply a diagnostic-outcome comparison that this single-arm study was not designed to perform.

### 2.2. CT System and Strategic Sequential TRO-CTA Protocol

**Figure 2:**
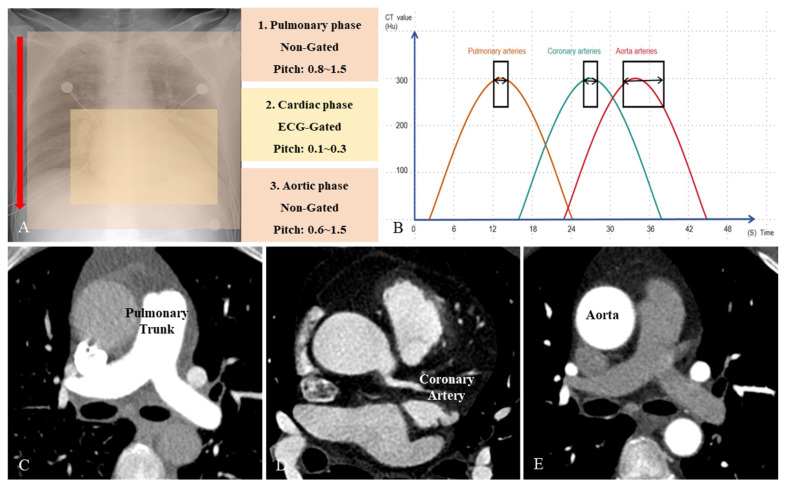
Principles of triple-rule-out CT angiography (TRO-CTA) using strategic sequential acquisition. (**A**) Overview of the sequential multiphase TRO-CTA protocol performed following a single contrast injection, comprising helical pulmonary phase acquisition, prospective ECG-gated axial coronary phase acquisition, and non-ECG-gated helical thoracic aortic phase acquisition using a wide–area detector CT system. (**B**) Phase-specific enhancement timing determined by individualized test-bolus analysis. Time–attenuation curves derived from ROIs in the pulmonary trunk and descending aorta demonstrate territory-dependent peak enhancement used to optimize acquisition timing for each phase. (**C**–**E**) Representative images obtained with sequential acquisition: (**C**) pulmonary phase image with diagnostic pulmonary arterial enhancement, (**D**) coronary phase (ECG-gated) image optimized for coronary visualization, and (**E**) aortic phase image optimized for thoracic aortic evaluation. CT: computed tomography; ECG: electrocardiography; ROI: region of interest.

All examinations were performed using a 320-row wide-area detector CT system (uCT 960+; United Imaging Healthcare, Shanghai, China). A structured sequential helical–axial–helical acquisition strategy was applied for tri-territory evaluation in the following order: pulmonary arteries, coronary arteries, and thoracic aorta (Figure 2). The pulmonary phase was prioritized to capture peak right-sided enhancement. The coronary phase was subsequently performed during physiologic transition to left-sided circulation, followed by thoracic aortic acquisition optimized for systemic arterial enhancement. Pulmonary and thoracic aortic phases were acquired using craniocaudal helical scanning. The coronary phase was obtained as a separate prospective ECG-gated axial acquisition centered on the heart, without table movement during the exposure window. All phases were acquired sequentially within a single examination session without patient repositioning. Given the emergency setting and the need to avoid treatment delay, no beta-blockers were administered. To maintain motion robustness under variable heart rate (HR) conditions, the prospective ECG acquisition window was intentionally set to 20–80% of the R-R interval, and the cardiac phase with the least motion artifact was selected for reconstruction and analysis.

### 2.3. Scan Parameters

For the coronary phase, a prospective ECG-gated axial acquisition was performed at 100 kVp with a fixed tube current of 180 mAs, using wide-detector coverage to enable whole-heart imaging within a single cardiac cycle. Pulmonary and thoracic aortic phases were acquired using helical scanning at 80 kVp, gantry rotation time 0.25 s, pitch 0.99, and automated tube current modulation (reference 66 mAs). The z-axis coverage was adapted according to the target vascular territory: 80 mm for pulmonary CTA to ensure broad thoracic coverage within a single breath-hold, and 40 mm for thoracic aortic CTA to limit overscanning and optimize spatial resolution in the distal thoracic aorta (Table 1). All acquisitions were performed in the craniocaudal direction.

### 2.4. Contrast Injection and Timing Strategy

Acquisition timing was individualized using a test-bolus technique. A 15-mL test injection of iodinated contrast material (Hexosure 350; iohexol 755 mg/mL, equivalent to 350 mg iodine/mL; Pharvis Pharmaceutical, Seoul, Republic of Korea) followed by 25 mL saline was administered at 5 mL/s. Time-to-peak enhancement was determined using regions of interest placed in the pulmonary trunk and descending thoracic aorta at the level of the bronchial bifurcation. For the pulmonary and thoracic aortic phases, scan initiation was defined as the measured time-to-peak enhancement of the respective target vessel plus 2 s to account for intravascular dispersion. The coronary phase was initiated approximately 10 s after completion of the pulmonary acquisition to allow physiologic transition from right-sided to left-sided circulation and to optimize systemic arterial enhancement. For the diagnostic acquisition, contrast material was administered at 1.2 mL/kg (injection rate 5 mL/s), followed by a saline flush.

### 2.5. Image Reconstruction and Post-Processing

All phases were reconstructed with a slice thickness and interval of 0.5 mm using a hybrid iterative reconstruction algorithm (KARL 3D; United Imaging Healthcare) at strength level 5. This level represents the routine clinical setting at our institution and was selected to balance noise reduction and spatial resolution. For coronary imaging, an experienced cardiac radiologist (24 years of experience) selected the phase with the least motion artifact within the reconstructed ECG window (20–80% R–R interval). Images were transferred to a dedicated workstation for objective and subjective image-quality analyses.

### 2.6. Objective Image Quality Analysis

**Figure 3:**
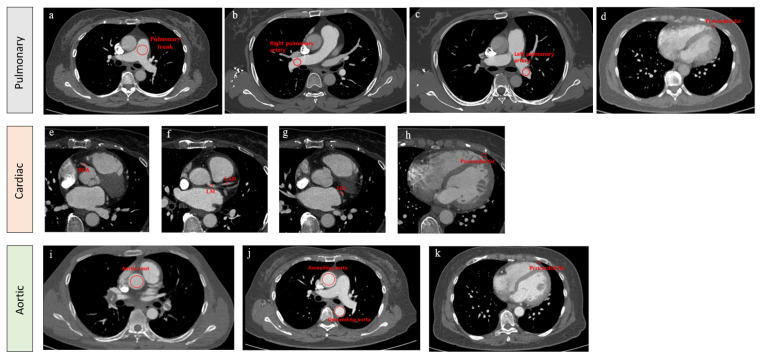
ROI placement for objective image-quality analysis. ROIs were placed in representative pulmonary, coronary, and aortic target vessels and in epicardial/pericardial fat for noise assessment. ROI: region of interest; RCA: right coronary artery; LM: left main; LAD: left anterior descending; LCx: left circumflex artery. Axial images demonstrate ROI locations used for attenuation, signal-to-noise ratio, and contrast-to-noise ratio measurements. Pulmonary phase: pulmonary trunk (**a**), right pulmonary artery (**b**), left pulmonary artery (**c**), and epicardial/pericardial fat (**d**). Coronary phase: right coronary artery (RCA) (**e**), left main (LM) and left anterior descending (LAD) arteries (**f**), left circumflex (LCx) artery (**g**), and epicardial/pericardial fat (**h**). Aortic phase: aortic root (**i**), ascending and descending thoracic aorta (**j**), and epicardial/pericardial fat (**k**).

Objective image quality was assessed by measuring mean attenuation (HU) and standard deviation (SD) using circular regions of interest (ROIs) placed in the proximal segments of the pulmonary trunk (PT), right and left pulmonary arteries (RPA, LPA), right coronary artery (RCA), left main (LM), proximal left anterior descending (LAD), proximal left circumflex (LCx), aortic root (AO), ascending aorta (AA), and descending thoracic aorta (DA) (Figure 3). ROIs were placed as large as anatomically possible within the vascular lumen while avoiding the vessel wall, calcification, motion artifact, and intraluminal abnormality. Approximate ROI sizes were 50–100 mm^2^ for the pulmonary and aortic territories and 3–5 mm^2^ for coronary arteries. Proximal segments were selected because they are primary diagnostic targets in TRO-CTA and provide standardized reference points for cross-patient and cross-study comparison. All measurements were performed by a single experienced observer to ensure consistency; repeated-measurement reproducibility was not assessed and is acknowledged as a limitation. A separate ROI was placed in epicardial fat at the level of the descending thoracic aorta to determine background attenuation and image noise. Image noise was defined as the SD of attenuation in epicardial fat. SNR was calculated as mean vessel attenuation divided by vessel SD, and CNR as (mean vessel attenuation − mean fat attenuation) divided by fat SD, in accordance with prior coronary and cardiothoracic CTA image-quality studies [24,25,26].

### 2.7. Subjective Image Quality Analysis

Two radiologists with experience in cardiac imaging independently evaluated subjective image quality. Image quality was graded using a predefined 5-point Likert scale adapted from prior coronary and cardiothoracic CTA studies. The scale incorporated vascular enhancement, motion artifacts, and image noise to determine diagnostic interpretability. Scores were defined as follows: 1, nondiagnostic; 2, poor; 3, acceptable; 4, good; and 5, excellent. A vascular territory was considered diagnostically interpretable if all major target vessels received a score of 3 or higher. The need for repeat TRO-CTA or additional contrast-enhanced CT due to insufficient image quality was recorded.

### 2.8. Radiation Dose Estimation

Radiation exposure parameters, including volume CT dose index (CTDIvol) and dose-length product (DLP), were automatically recorded by the CT system for each acquisition phase and for the total examination. DLP was reported as the primary standardized dose metric. Effective dose was not calculated because chest-specific conversion factors vary according to patient characteristics, scan range, ECG synchronization, and protocol design, and because DLP allows direct comparison of scanner-reported radiation output across the three acquisition phases.

### 2.9. Statistical Analysis

Statistical analyses were performed using SPSS (version 26.0; IBM Corp., Armonk, NY, USA), GraphPad Prism (version 9.0; GraphPad Software, Boston, MA, USA), and independent R (version 4.x; R Foundation for Statistical Computing, Vienna, Austria) and Python (version 3.x; Python Software Foundation)-based verification. Continuous variables are presented as mean ± standard deviation (SD) with 95% confidence intervals (CIs) for key objective image-quality parameters, and categorical variables are presented as frequencies and percentages. Territory-level attenuation values were calculated as the patient-level mean of the predefined pulmonary, coronary, or aortic vascular measurements. Paired *t*-tests were used for pairwise comparisons among pulmonary, coronary, and aortic territory-level attenuation values. Qualitative (subjective) image-quality scores are summarized as medians with interquartile ranges (IQRs). Interobserver agreement for subjective image-quality scoring was assessed using linearly weighted kappa statistics and interpreted as follows: 0.00–0.20, slight; 0.21–0.40, fair; 0.41–0.60, moderate; 0.61–0.80, good; and 0.81–1.00, excellent agreement. A two-sided *p* value < 0.05 was considered statistically significant.

## 3. Results

### 3.1. Baseline Characteristics and Clinical Findings

The final quantitative cohort included 71 patients (mean age, 66.6 ± 17.0 years; 33 women) who underwent TRO-CTA for evaluation of undifferentiated acute chest pain in the ED. Baseline characteristics are summarized in Table 2. The mean HR during acquisition was 79.2 ± 16.3 bpm, reflecting the absence of routine pharmacologic HR control. Nineteen of 90 initially identified patients (21.1%) were excluded from the primary tri-territory quantitative analysis because of incomplete evaluability: patient motion-related artifact (*n* = 15), clinically significant arrhythmia precluding reliable ECG-gated acquisition (*n* = 2), and inability to cooperate due to altered mental status (*n* = 2). Findings in the final cohort were distributed across the three target disease categories: ACS (*n* = 10), APE (*n* = 8), and AAS (n = 4). Significant coronary artery stenosis without evidence of ACS was identified in an additional 7 patients. Alternative diagnoses included pneumonia (*n* = 7), pleuritis (*n* = 1), pericarditis (*n* = 2), myocarditis (*n* = 3), and stress-induced cardiomyopathy (*n* = 1). Twenty-eight patients (39.4%) had no acute cardiothoracic cause identified. Contrast material was administered according to the weight-adjusted protocol (1.2 mL/kg), with a mean volume of 75.6 ± 15.8 mL.

### 3.2. Objective Image Quality

**Figure 4:**
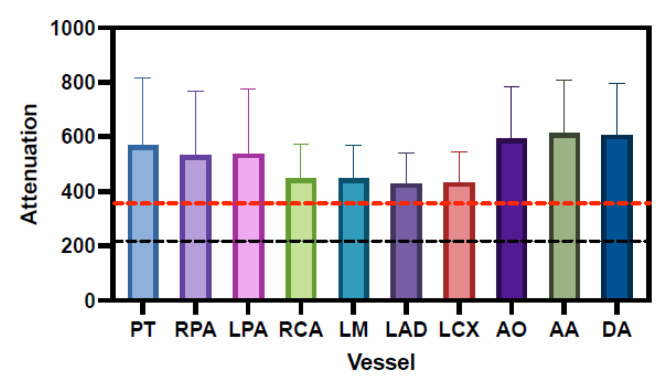
Target vascular attenuation achieved by sequential TRO-CTA. Bars show mean attenuation with error bars indicating ±1 SD. Dashed lines denote prespecified diagnostic attenuation thresholds for coronary and pulmonary/aortic vasculature. Bar graphs show mean vascular attenuation (HU) with error bars indicating ±1 standard deviation. Dashed horizontal lines denote prespecified diagnostic attenuation thresholds for coronary arteries (≥350 HU) and pulmonary/aortic vasculature (≥210 HU). PA: pulmonary artery; RCA: right coronary artery; LM: left main coronary artery; LAD: left anterior descending coronary artery; LCx: left circumflex coronary artery; AO: aortic root; AA: ascending aorta; DA: descending aorta.

**Figure 5:**
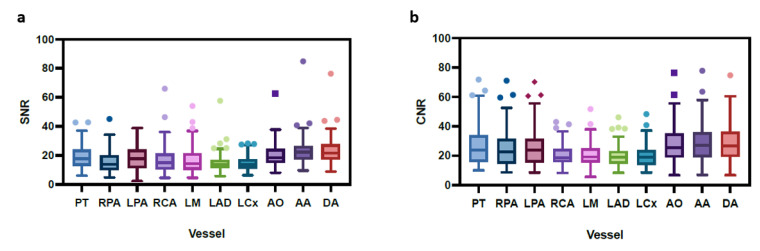
Objective image-quality metrics across vascular territories. Box-and-whisker plots of (**a**) signal-to-noise ratio (SNR) and (**b**) contrast-to-noise ratio (CNR) for pulmonary, coronary, and aortic territories. Center lines indicate medians, box limits indicate interquartile ranges (IQRs), whiskers represent 1.5 × IQR, circles indicate mild outliers (1.5–3 × IQR beyond box limits), diamonds indicate extreme outliers (>3 × IQR beyond box limits), and squares indicate mean values. SNR: signal-to-noise ratio; CNR: contrast-to-noise ratio; PT: pulmonary trunk; RPA: right pulmonary artery; LPA: left pulmonary artery; RCA: right coronary artery; LM: left main coronary artery; LAD: left anterior descending coronary artery; LCx: left circumflex coronary artery; AO: aortic root; AA: ascending aorta; DA: descending aorta.

Phase-appropriate vascular enhancement was achieved across all territories (Figure 4). Territory-level mean attenuation was 546.7 ± 237.8 HU (95% CI, 490.4–603.0) for the pulmonary arteries, 438.8 ± 113.9 HU (95% CI, 411.9–465.8) for the coronary arteries, and 604.3 ± 190.9 HU (95% CI, 559.2–649.5) for the thoracic aorta. Pairwise comparisons showed significant differences among territories (pulmonary vs. coronary: mean difference, 107.9 HU; 95% CI, 69.7–146.1; *p* < 0.001; pulmonary vs. aortic: mean difference, −57.7 HU; 95% CI, −93.3 to −22.0; *p* = 0.002; coronary vs. aortic: mean difference, −165.5 HU; 95% CI, −192.8 to −138.3; *p* < 0.001). Despite these statistically significant differences, all territories exceeded the predefined diagnostic attenuation thresholds. SNR and CNR were also assessed across all vascular territories (Figure 5); the aortic segments demonstrated the highest SNR and CNR values, followed by the pulmonary arteries, while the coronary arteries showed comparatively lower values consistent with their smaller caliber. Vessel-level objective image-quality values with 95% CIs are summarized in Table 3.

### 3.3. Subjective Image Quality and Diagnostic Interpretability

**Figure 6:**
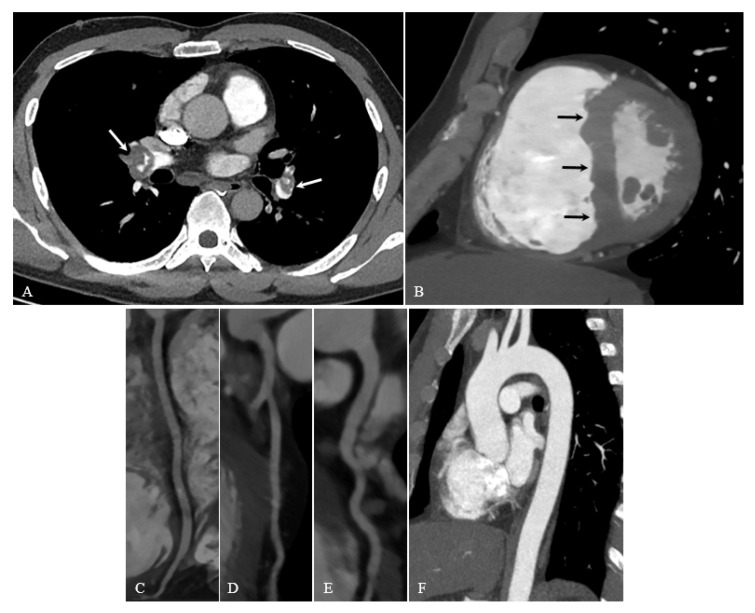
Representative TRO-CTA in acute pulmonary embolism. A 45-year-old man with acute dyspnea and chest discomfort. (**A**) Pulmonary phase image shows filling defects in bilateral segmental and subsegmental pulmonary arteries (arrows). (**B**) Short-axis coronary phase (ECG-gated) image demonstrates right ventricular dilatation and interventricular septal flattening (arrows). (**C**–**E**) Curved multiplanar reformations (MPR) coronary phase (ECG-gated) images show normal RCA (**C**), LAD (**D**), and LCx (**E**) without stenosis. (**F**) Oblique sagittal MPR aortic phase image shows no acute aortic abnormality. RCA: right coronary artery; LAD: left anterior descending coronary artery; LCx: left circumflex artery; MPR: multiplanar reformation.

**Figure 7:**
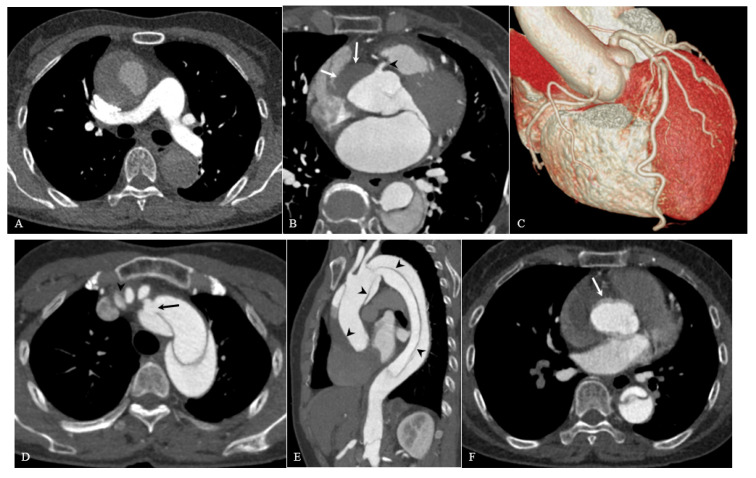
Representative TRO-CTA in acute aortic syndrome. A 64-year-old woman with acute chest pain. (**A**) Pulmonary phase image demonstrates Stanford type A aortic dissection involving the ascending and descending thoracic aorta with preserved pulmonary arterial enhancement. (**B**) Coronary phase (ECG-gated) image shows extension of the false lumen into the right sinus of Valsalva (arrows) without significant right coronary artery obstruction. (**C**) Volume-rendered coronary phase (ECG-gated) image demonstrates no significant stenosis in the epicardial coronary arteries. (**D**,**E**) Aortic phase images demonstrate the entry tear (arrow), extension to the brachiocephalic artery (arrowhead), and the intimal flap. (**F**) Aortic phase axial image demonstrates double contouring at the aortic root due to pulsation-related motion artifact on non-ECG-gated acquisition.

**Figure 8:**
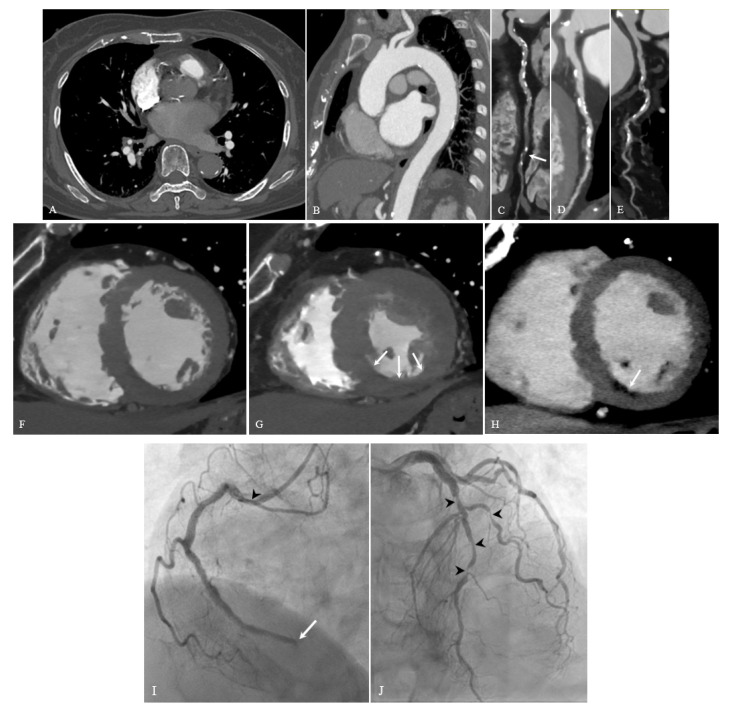
Representative TRO-CTA in acute coronary syndrome. A 79-year-old woman with acute chest pain. (**A**) Pulmonary phase image shows no pulmonary embolism. (**B**) Aortic phase image demonstrates atherosclerotic change in the descending thoracic aorta without acute aortic syndrome. (**C**–**E**) Curved multiplanar reformations (MPR) coronary phase (ECG-gated) images demonstrate severe coronary calcification in the RCA (**C**), LAD (**D**), and LCx (**E**), with significant stenosis of the distal RCA (arrow). (**F**,**G**) Short-axis coronary phase (ECG-gated) images demonstrate regional wall motion abnormality in the mid-inferior and mid-inferoseptal LV walls (arrows). (**H**) Delayed enhancement image demonstrates subendocardial hypoenhancement (arrow) consistent with myocardial infarction. (**I**,**J**) Invasive coronary angiography confirms complete occlusion of the distal RCA (arrow) and significant stenosis in additional segments (arrowheads). MPR: multiplanar reformation; LV: left ventricle; RCA: right coronary artery; LAD: left anterior descending coronary artery; LCx: left circumflex artery.

Interobserver agreement was excellent for pulmonary and coronary phases and good for the aortic phase (kappa = 0.92, 0.90, and 0.80, respectively; all *p* < 0.001). Median subjective image quality scores were 5 (IQR, 4–5) for the pulmonary phase, 4.5 (IQR, 4–5) for the coronary phase, and 4 (IQR, 4–5) for the aortic phase. All examinations in the qualitative assessment cohort were considered diagnostically interpretable in pulmonary, coronary, and aortic territories (territory score ≥ 3). The absence of routine beta-blocker administration did not prevent diagnostic coronary interpretability in the evaluable cohort; however, patients with clinically significant arrhythmia that precluded reliable ECG-gated acquisition were excluded from the primary quantitative analysis. Representative cases are shown in Figure 6, Figure 7 and Figure 8.

### 3.4. Radiation Doses

The mean total DLP was 461.5 ± 122.5 mGy·cm. Phase-specific DLP values were 65.2 ± 13.6 mGy·cm for the pulmonary phase, 337.2 ± 118.0 mGy·cm for the ECG-gated coronary phase, and 59.1 ± 16.5 mGy·cm for the thoracic aortic phase, indicating that the coronary acquisition accounted for approximately 73% of the total radiation output. This distribution reflects the intentionally broad 20–80% R-R acquisition window used to preserve coronary motion robustness under emergency conditions without routine HR control.

## 4. Discussion

This study evaluated the technical feasibility and image quality of a sequential helical–axial–helical TRO-CTA protocol in patients presenting to a tertiary ED with undifferentiated acute chest pain. The principal finding is that territory-specific vascular enhancement of the pulmonary arteries, coronary arteries, and thoracic aorta can be consistently achieved within a single structured examination using individualized contrast timing, even without routine HR-lowering medication. The revised interpretation of this study is deliberately conservative: the results support technical feasibility and image-quality consistency, but they do not establish diagnostic superiority or improved clinical outcomes compared with conventional TRO-CTA. Direct prospective head-to-head comparison remains necessary.

Comparison with previous studies should be interpreted cautiously because the present study did not include a direct comparator arm. Earlier TRO-CTA protocols have pursued dose reduction or contrast optimization using single-acquisition strategies, dual-source high-pitch scanning, low-kV imaging, iterative reconstruction, or wide-detector systems [16,17,18,22,27,28,29]. In contrast, the present protocol was designed primarily to provide phase-specific pulmonary, coronary, and aortic enhancement under real-world emergency conditions rather than to prove superiority over any single conventional approach.

The clinical implication of this technical approach is that a structured sequential TRO-CTA protocol may help ED teams evaluate ACS, APE, and AAS within a single coordinated workflow when all three diagnoses remain clinically plausible. In particular, consistent enhancement across all three vascular territories may reduce the need for repeat contrast-enhanced imaging caused by inadequate bolus timing and may improve diagnostic confidence in patients with overlapping presentations. However, this remains an inferential implication rather than a proven outcome benefit, because diagnostic accuracy, time to diagnosis, downstream management, and patient outcomes were not formally assessed in the present study.

Radiation exposure remains an important consideration. The mean total DLP of 461.5 mGy·cm is within the range of clinically used prospective ECG-gated TRO-CTA protocols but is not negligible, and most of the dose was attributable to the coronary phase [22,27,28,29]. This tradeoff was accepted to maintain motion robustness in emergency patients with variable HRs and no routine beta-blocker administration. Future protocol refinements should focus on coronary-phase dose reduction, including narrower ECG acquisition windows in stable lower-HR patients, lower tube voltage in appropriate body habitus, automated tube-current modulation, model-based or deep learning reconstruction, and artificial intelligence (AI)-assisted phase selection.

Coronary imaging without routine HR control is both a strength and a limitation. It reflects real-world ED practice, where beta-blocker administration may be contraindicated or may delay imaging, and the combination of 16-cm wide-detector axial coverage, 0.25-s rotation, a broad 20–80% R-R acquisition window, and best-phase reconstruction enabled diagnostic coronary interpretability in technically analyzable cases despite a mean HR of 79.2 ± 16.3 bpm. Nevertheless, performance in sustained tachycardia (>100 bpm) or clinically significant arrhythmia is expected to be more variable, and patients with arrhythmia that precluded reliable ECG-gated acquisition were excluded from the primary quantitative analysis.

Study strengths include the use of a consecutive ED cohort, a clearly defined physiology-based acquisition sequence, individualized test-bolus timing, standardized quantitative ROI placement, territory-specific objective and subjective image-quality assessment, independent two-reader subjective evaluation, and implementation in a tertiary cardiovascular emergency referral environment.

Future directions include prospective multicenter validation, direct comparison with conventional single-acquisition TRO-CTA, and integration of AI-assisted reconstruction and analysis. AI-based tools may support bolus-timing prediction, motion-robust coronary reconstruction, low-dose reconstruction, automated vessel segmentation, ROI standardization, automated image-quality assessment, and triage of positive findings [20,21,24].

A relatively high exclusion rate from the primary tri-territory quantitative analysis (19/90; 21%) may have led to overestimation of objective image-quality metrics. This should be interpreted carefully. The excluded cases reflect real-world ED limitations such as motion, arrhythmia, and impaired cooperation rather than diagnosis-based or outcome-based selection. We did not add a detailed excluded-patient clinical comparison because such analysis would be beyond the technical feasibility scope of this study and could imply a diagnostic-outcome investigation that was not performed. Therefore, the reported quantitative metrics should be understood as performance in technically analyzable tri-territory examinations, and prospective multicenter studies are needed to determine all-comer diagnostic yield and generalizability.

Finally, individualized test-bolus timing may increase workflow complexity in high-throughput emergency settings. Although the protocol was feasible in our tertiary ED practice, future studies should formally evaluate scan-room time, technologist workflow, and operational reproducibility. Simplified fixed-delay or bolus-tracking adaptations may be useful where individualized test-bolus expertise is not readily available.

## 5. Conclusions

In conclusion, a structured sequential TRO-CTA protocol based on physiology-driven acquisition timing is technically feasible in a tertiary emergency setting and provides consistent tri-territory image quality in technically analyzable examinations. This approach may represent a practical alternative to conventional single-acquisition TRO strategies when ACS, APE, and AAS remain simultaneously plausible, but its diagnostic and clinical impact requires prospective comparative validation.

## Figures and Tables

**Table 1:** Scan parameters for the strategic sequential TRO-CTA protocol.

Parameters	Pulmonary Phase	Cardiac Phase	Thoracic Aorta Phase
Scan method	Helical	Axial	Helical
Reconstruction technique	HIR	HIR	HIR
Tube voltage (kVp)	80	100	80
Tube current (mA)	Auto (Ref. 66 mAs)	180	Auto (Ref. 66 mAs)
Collimation (mm)	0.5 × 320	0.5 × 320	0.5 × 320
Rotation time (s)	0.25	0.25	0.25
Slice thickness (mm)	0.5	0.5	0.5
Pitch	0.99	-	0.99
R-R interval	-	20–80	-

TRO-CTA: triple-rule-out computed tomography angiography; HIR: hybrid iterative reconstruction.

**Table 2:** Characteristics of patients undergoing the strategic sequential TRO-CTA protocol.

Criteria	Patients Enrolled (*n* = 71)
Age (years)	66.6 ± 17.0
Female (n,%)	33 (46.5%)
Body mass index (kg/m^2^)	24.0 ± 3.7
HR (bpm)	79.2 ± 16.3
Main clinical characteristics	
COPD	3 (4%)
Hypertension	35 (49%)
Diabetes Mellitus	20 (28%)
NYHA (class ≥ 2)	20 (28%)
Disease distribution	
APE	8 (11.3%)
AAS	4 (5.6%)
ACS	10 (14.1%)
Pneumonia	7 (9.9%)
Pleuritis	1 (1.4%)
Pericarditis	2 (2.8%)
Myocarditis	3 (4.2%)
Stress-induced cardiomyopathy	1 (1.4%)
Significant coronary stenosis without ACS	7 (9.9%)
Non-specific findings	28 (39.4%)
Volume of contrast media (mL)	75.6 ± 15.8
Dose length product (mGy·cm)	
Pulmonary	65.24 ± 13.59
Cardiac	337.2 ± 118.0
Aortic	59.07 ± 16.53
Total	461.52 ± 122.52

Data are presented as mean (standard deviation) or frequency (percentage). TRO-CTA: triple-rule-out computed tomography angiography; bpm: beat per minute; COPD: chronic obstructive pulmonary disease; NYHA: New York Heart Association; APE: acute pulmonary embolism; AAS: acute aortic syndrome; ACS: acute coronary syndrome.

**Table 3:** Objective and subjective image quality metrics.

Criteria	Patient (n = 71)			
	Attenuation (HU) Mean ± SD (95% CI)	SNR Mean ± SD (95% CI)	CNR Mean ± SD (95% CI)	Subjective Image Score
PT	569.5 ± 245.8 (95% CI, 511.3–627.7)	19.16 ± 8.62 (95% CI, 17.12–21.20)	26.74 ± 13.77 (95% CI, 23.48–29.99)	5 (IQR, 4–5)
RPA	532.8 ± 236.1 (95% CI, 476.9–588.7)	15.60 ± 7.50 (95% CI, 13.82–17.37)	25.23 ± 13.23 (95% CI, 22.09–28.36)	
LPA	537.8 ± 239.5 (95% CI, 481.1–594.5)	18.13 ± 8.53 (95% CI, 16.11–20.15)	25.57 ± 13.47 (95% CI, 22.38–28.76)	
RCA	447.3 ± 125.6 (95% CI, 417.6–477.1)	17.22 ± 9.67 (95% CI, 14.93–19.51)	20.09 ± 7.62 (95% CI, 18.28–21.89)	4.5 (IQR, 4–5)
LM	446.0 ± 123.6 (95% CI, 416.8–475.3)	17.03 ± 9.37 (95% CI, 14.81–19.25)	20.11 ± 7.94 (95% CI, 18.23–21.99)	
LAD	429.3 ± 113.7 (95% CI, 402.4–456.2)	15.13 ± 7.20 (95% CI, 13.42–16.83)	19.53 ± 7.83 (95% CI, 17.67–21.38)	
LCx	432.6 ± 114.8 (95% CI, 405.4–459.8)	14.81 ± 5.23 (95% CI, 13.57–16.04)	19.63 ± 7.92 (95% CI, 17.76–21.50)	
Aortic root	593.7 ± 191.2 (95% CI, 548.4–639.0)	20.11 ± 8.28 (95% CI, 18.15–22.07)	29.17 ± 13.22 (95% CI, 26.04–32.30)	4 (IQR, 4–5)
Ascending aorta	612.2 ± 193.8 (95% CI, 566.3–658.0)	23.85 ± 10.53 (95% CI, 21.36–26.35)	29.96 ± 13.42 (95% CI, 26.78–33.14)	4 (IQR, 4–5)
Descending aorta	607.2 ± 191.0 (95% CI, 562.0–652.4)	23.22 ± 10.18 (95% CI, 20.81–25.63)	29.77 ± 13.27 (95% CI, 26.63–32.91)	

Data are presented as mean ± standard deviation (95% CI) or median (interquartile range), as appropriate. SNR: signal-to-noise ratio; CNR: contrast-to-noise ratio; PT: pulmonary trunk; RPA: right pulmonary artery; LPA: left pulmonary artery; RCA: right coronary artery; LM: left main coronary artery; LAD: left anterior descending artery; LCx: left circumflex artery.

## Data Availability

The data presented in this study are available from the corresponding author upon reasonable request. The data are not publicly available due to institutional privacy regulations and ethical restrictions.

## Abbreviations

The following abbreviations are used in this manuscript:

ACS	Acute coronary syndrome
APE	Acute pulmonary embolism
AAS	Acute aortic syndrome
CTA	Computed tomography angiography
TRO-CTA	Triple-rule-out computed tomography angiography
ED	Emergency department
ECG	Electrocardiography
HR	Heart rate
HU	Hounsfield unit
ROI	Region of interest
SNR	Signal-to-noise ratio
CNR	Contrast-to-noise ratio
CTDIvol	Volume computed tomography dose index
DLP	Dose-length product
IQR	Interquartile range
NYHA	New York Heart Association
COPD	Chronic obstructive pulmonary disease
RCA	Right coronary artery
LM	Left main coronary artery
LAD	Left anterior descending coronary artery
LCx	Left circumflex coronary artery
PT	Pulmonary trunk
RPA	Right pulmonary artery
LPA	Left pulmonary artery
AO	Aortic root
AA	Ascending aorta
DA	Descending thoracic aorta
MPR	Multiplanar reformation
